# Hope, optimism and promises for 2021

**DOI:** 10.1016/j.lanepe.2021.100043

**Published:** 2021-02-05

2020 has been a momentous year in which every single person, regardless of class, gender, or ethnicity, was affected directly or indirectly by the COVID-19 pandemic, which at the time of writing has claimed more than 2 million lives. Despite the turmoil caused, the pandemic also brought about the potential to catalyse new developments in medicine and public health. The rollout of vaccines against severe acute respiratory syndrome coronavirus 2 (SARS-CoV-2) a year into the pandemic is a remarkable feat—the first of its kind to be achieved by humanity and science. Additionally, the transition from a complacent response at the start of the pandemic to a more vigilant and rapid response to limit the spread of new virus variants B.1.1.7 and 501Y.V2 from the UK and South Africa, respectively, since mid-December, 2020, testifies that lessons have been learned from earlier mistakes.

The road ahead in 2021 is more hopeful and optimistic, although not an easy one. There are three new pertinent challenges. First and foremost, mass vaccination—with the constraints of time, resources, manufacturing capacity, and delivery logistics. Second, vaccine scepticism, highly pronounced in the Europe region, and largely fuelled by misinformation. Although vaccine sceptics constitute a minority in the community, it is important to prevent the proliferation of such sentiment. Third, bridging the inequality divide that the pandemic has magnified between the digital class (who can work from home) and non-digital class (who cannot work from home and are hit hardest economically). Above all, this pandemic has exposed the fragility of health-care systems and the consequences of a lack of unity among European countries in responding to the pandemic in unison**.** This pandemic has therefore been a wake-up call, highlighting the need for a foundation and platform of convergence for scientists, clinicians, and policy makers from diverse fields, to lend a new voice to discuss and contextualise clinical, health, and policy matters on pressing issues in Europe such as COVID-19.

To help meet the health and clinical challenges in the Europe region, we are launching *The Lancet Regional Health – Europe*, an online open access journal, part of *The Lancet*'s global initiative for regional health. The journal's aim is to provide a natural home and distinctive voice for all clinicians and health researchers, to publish high quality work that can advance their field of research or influence the health policies of countries in the WHO Europe region. All areas spanning the whole of clinical and public health spectrum are included within the scope. The journal's core mission is to publish content from all corners of Europe, to disseminate key findings on clinical practice, public health, and policies, and to raise awareness and prompt public debate with the ultimate goal of improving health outcomes and narrowing health inequality gaps in the region. In keeping with *The Lancet* legacy, the editorial and review process will be honest and constructive, and we will strive to provide the highest possible level of editorial service. Through our Editorial, Review, Viewpoint, Comment, and Series, we aim to bring together research scientists, clinicians, policy experts, authors, and reviewers to lead the debate on priority areas of concern in Europe. Selected Research papers will also be accompanied by a linked Commentary to contextualise the original findings for a broader audience.

The journal's first issue is testimony to our commitment to represent voices from all corners of Europe, diversity in topic, and our efforts towards bridging the health inequality gap. This inaugural issue features content from different European regions, including the UK, Switzerland, the Netherlands, Faroe Islands, Uzbekistan, and Turkey. The articles cover a wide spectrum of topics, including attitudes towards COVID-19 vaccines, socioeconomic deprivation and COVID-19 mortality, European first responder systems, payer status impact on cardiac surgery, travel-related infections in Europe, and Turkey's non-communicable disease policy targets. Compelling commentaries on the need for a European Health Union, and the impact of climate change on vector-borne diseases in Europe are representative of our mission to highlight the pulse of the region. We are grateful to all the authors who entrusted us with their work and continue to do so. We also thank *The Lancet* family journals for the support in informing authors about *The Lancet Regional Health – Europe* as a natural home for their papers. Last but not least, we thank our Advisory Board members for being on board with us from the journal inception, and for believing in our mission to provide a common voice for Europe.

The COVID-19 pandemic is not the first, and will not be the last time that humanity is confronted with such a cross-border health challenge. The development of COVID-19 vaccines is a fine example of a medical marvel that can be achieved when there is a common will across nations and different sectors: science, technology, industry, and politics. We hope that *The Lancet Regional Health – Europe* will be a strong platform that brings together all stakeholders and fosters collaboration and unity among European countries to champion and support science and health in Europe.

*The Lancet Regional Health – Europe*

